# Use of cuff tear arthroplasty head prosthesis for rotator cuff arthropathy treatment in elderly patients with comorbidities

**DOI:** 10.1590/S1679-45082016AO3372

**Published:** 2016

**Authors:** Cassiano Diniz Carvalho, Carlos Vicente Andreoli, Alberto de Castro Pochini, Benno Ejnisman

**Affiliations:** 1 Centro de Traumatologia do Esporte, Escola Paulista de Medicina, Universidade Federal de São Paulo, São Paulo, SP, Brazil.

**Keywords:** Rotator cuff, Joint diseases, Arthroplasty, Hemiarthroplasty, Arthroplasty, replacement

## Abstract

**Objective:**

To evaluate the clinical and functional behavior of patients undergoing cuff tear arthroplasty at different stages of the disease.

**Methods:**

Cuff tear arthroplasty hemiarthroplasties were performed in 34 patients with rotator cuff arthropathy and associated comorbidities, classified according to Seebauer. The mean age was 76.3 years, and the sample comprised 23 females (67.6%) and 11 males (32.4%). The mean follow-up period was 21.7 months, and evaluations were performed using the Visual Analog Scale for pain and the Constant scale.

**Results:**

There were no statistically significant differences in the mean reduction in the Visual Analog Scale or in the Constant scale increase between the female and male groups. The variation between the pre- and postoperative Visual Analog Scale and Constant scale evaluations was significant. There was also no statistically significant difference between the Seebauer classification groups regarding the mean Visual Analog Scale reduction, or the mean Constant scale increase.

**Conclusion:**

Cuff tear arthroplasty shoulder hemiarthroplasty is a good option for rotator cuff arthropathy in patients with comorbidities.

## RESUMO

**Objetivo:**

Avaliar o comportamento clínico e funcional dos pacientes submetidos à artroplastia do tipo *cuff tear arthroplas*ty para o tratamento da artropatia do manguito rotador em diferentes estágios da afecção.

**Métodos:**

Foram realizadas 34 hemiartroplastias do tipo *cuff tear arthroplas*ty em 34 pacientes com artropatia do manguito rotador e comorbidades associadas, classificadas de acordo com Seebauer. A média de idade foi de 76,3 anos, sendo 23 pacientes do sexo feminino (67,6%) e 11 do sexo masculino (32,4%). O seguimento médio foi de 21,7 meses e a avaliação foi realizada por meio da Escala Visual Analógica da dor e pela escala de Constant.

**Resultados:**

Não houve diferença estatisticamente significante entre os grupos feminino e masculino, tanto nas médias de redução na Escala Visual Analógica quanto nas de aumento na escala de Constant. A variação entre as avaliações da Escala Visual Analógica e da escala de Constant pré e pós-operatórias foi significante. Não houve diferença estatisticamente significante entre os grupos de classificação de Seebauer quanto às médias de redução na Escala Visual Analógica e nem quanto às médias de aumento na escala de Constant, e não houve casos de infecção.

**Conclusão:**

A hemiartroplastia do ombro tipo *cuff tear arthroplas*ty é boa uma opção nos pacientes com artropatia do manguito rotador, especialmente em pacientes com comorbidades.

## INTRODUCTION

The term “degenerative arthropathy of the rotator cuff” was first described by Neer, in 1983, and means the collapse of the glenohumeral joint secondary to a massive chronic lesion of the rotator cuff, causing insufficiency of the rotator cuff, rising (cranialization) of the humeral head, joint destruction, synovial fluid alterations, subchondral cysts, flattening of the greater tubercle, osteophytes, acetabularization of the coracoacromial arch, and osteopenia;^(1-4)^ the first three changes were present in all patients.^(5)^

Shoulder arthropathy occurs more frequently in female patients aged over 60 years and is manifested with pain, crepitus, and decreased range of motion.^(4)^ Despite the fact that many patients present with biomechanical alterations due to the rotator cuff lesion, not all of them will developed degenerative arthropathy. The exact etiology of arthropathy remains uncertain.^(5)^ Neer et al. initially proposed that biomechanical and nutritional alterations resulting from a rotator cuff lesion would lead to joint degeneration and humeral head osteopenia. However, this theory did not explain why not all patients with cuff lesions develop arthropathy.^(3)^

Some hypotheses have been raised to clarify the etiology. Among them, the Milwaukee shoulder was used to describe the association between massive lesion of the rotator cuff, glenohumeral arthropathy, and recurrent effusion of fluid through the acromioclavicular joint (geyser sign). This theory defends that the accumulation of hydroxyapatite crystals in the capsule and joint cartilage would release them into the synovial fluid. Once phagocyted by synovial fluid cells, the crystals would stimulate the production of proteolytic enzymes, including collagenase and protease. Finally, these enzymes would be responsible for the destruction of the joint, capsule and cuff.^(6)^

**Figure 1:**
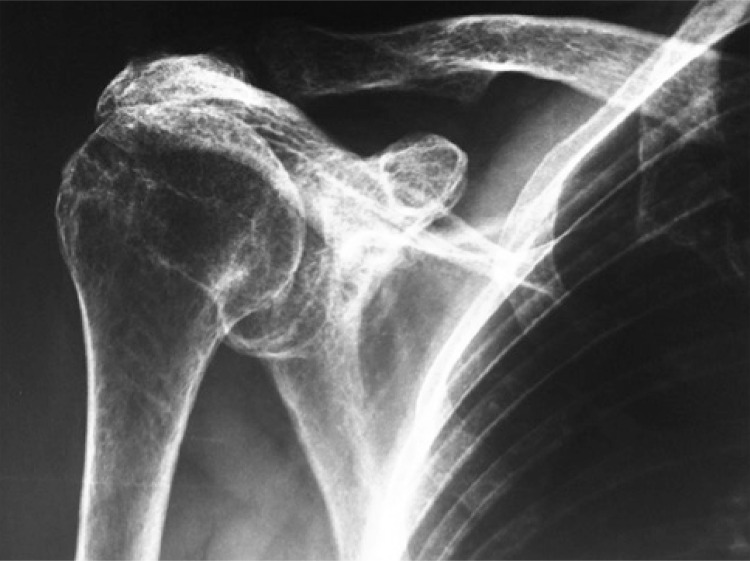
Radiograph of a patient classified as Seebauer IIA

The classification of Hamada divides the massive cuff lesions into five stages,^(7)^ whereas the Seebauer classification is radiographically correlated with the pathology of the rotator cuff. In the Seebauer IA class, the humeral head remains centered in the glenoid; in IB, it is medially dislocated towards the glenoid; in IIA, it is dislocated upwardly (Figure 1); and in IIB, it is dislocated anterosuperiorly, triggering loss of the coracoacromial arch.^(2)^

The initial treatment should be conservative, with modification of activities, oral analgesics, physical therapy, and intra-articular injection of corticoids. The latter is initially effective, but its repeated use should be discouraged due to decreased efficacy and the possible risk of infection.^(5)^

Replacement arthroplasty options include cuff tear arthroplasty (CTA^®^) and reverse prosthesis. The CTA^®^ prosthesis is used when the arthropathy did not compromise the stability of the glenohumeral joint, erosion of the glenoid is partial, and the coracoacromial arch is intact. It is a partial prosthesis with a larger humeral head in order to provide greater contact with the coracoacromial arch, allowing improvement of the lever arm of the deltoid muscle in arm elevation movement.^(1,6,8)^

From this perspective, the present study aims to present a procedure with less morbidity as compared to reverse prosthesis, and with good results in patients requiring less invasive surgery with shorter operative time.

## OBJECTIVE

To evaluate the clinical and functional behavior of patients submitted to cuff tear arthroplasty to treat rotator cuff arthropathy at different stages of the condition.

## METHODS

Thirty-four CTA^®^ hemiarthroplasties were performed in 34 patients with rotator cuff arthropathy, diagnosed by history, physical examination, radiographs, and magnetic resonance imaging of the shoulder, using the Seebauer classification. Six cases were type IA, 12 were IB, 8 were IIA, and 8 were IIB. All patients were operated on by the Shoulder Group of the Trauma and Sports Center of the *Universidade Federal de São Paulo*, between January 2007 and December 2012.

The inclusion criteria for these studies were patients with arthropathy of the rotator cuff classified as Seebauer IA, IB, IIA, and IIB, who had some associated comorbidity that could increase surgical morbidity. Excluded were patients that improved after conservative treatment, presented no comorbidities, with deltoid insufficiency, prior infection, and peripheral neurological lesion. In cases classified as IIB, surgery was indicated to relieve pain, and all patients presented with comorbidities (Table 1).

**Table 1:** Patient distribution

Patients	Sex	Age	Seebauer	Comorbidities	Preoperative VAS	Postoperative VAS	Preoperative Constant scale	Postoperative Constant scale
CBR	F	71	IA	Cardiopathy	9	2	50	70
MERO	F	72	IB	1 MR	7	1	35	64
CHERT	M	70	IB	Stroke	8	0	46	60
GCMS	F	81	IIB	2 MR	9	2	10	40
TOWC	F	74	IIA	1 MR	6	0	37	55
ASDR	M	70	IB	Cardiopathy	8	1	40	60
NAM	F	85	IIB	1 MR	7	1	7	23
FCHJ	F	78	IIB	Parkinson’s Disease	9	2	2	15
ZSER	M	80	IIB	Alzheimer’s disease	6	3	15	49
CBS	F	80	IB	Cardiopathy	7	1	30	60
RS	F	75	IA	Parkinson’s Disease	8	0	45	75
PRTY	F	78	IIA	1 MR	7	1	40	64
LEOC	M	83	IB	AVC	9	1	40	60
FB	F	71	IA	Cardiopathy	6	1	60	83
SRO	F	76	IB	DM	9	2	36	75
MUO	M	72	IA	Cardiopathy	7	0	46	70
ACM	F	80	IIA	Alzheimer’s disease	8	1	30	60
AZD	F	82	IIB	Mild stroke	7	2	10	33
BE	M	78	IB	Lung disease	9	1	45	65
GCM	F	71	IB	2 MR	8	0	45	65
AP	F	74	IIA	Cardiopathy	8	2	35	70
PB	M	78	IIA	Cardiopathy	7	1	35	60
MC	F	79	IIB	Cardiopathy	7	0	7	34
OS	F	80	IB	Alzheimer’s disease	9	1	35	60
ETO	M	73	IA	DM	7	0	42	64
ADE	F	74	IB	1 MR	8	3	45	64
RTZS	M	78	IIB	1 MR	7	2	15	34
EMA	F	79	IIA	DM	9	1	36	55
VA	M	83	IB	Stroke	7	1	40	75
TCM	F	77	IIB	Cardiopathy	9	0	9	32
OCM	F	72	IB	2 MR	8	3	30	55
MCCA	M	71	IIA	Cardiopathy	7	2	30	45
SFJO	F	80	IIB	Lung disease	7	1	13	37
SEC	F	68	IIA	Cardiopathy	9	1	30	60

F: female; M: male; VAS: Visual Analog Scale; AVC: *acidente vascular cerebral*; DM: *diabetes mellitus*; MR: myocardial revascularization.

The conservative treatment instituted was the use of medication, such as corticoids and analgesics, as well as appropriate physical therapy for six months. The mean age was 76.3 years (range of 68-85 years), and 23 patients were female (67.6%).

The mean follow-up was 21.7 months (range of 8 months - 6 years). The evaluation was done by means of the Visual Analog Scale (VAS) and the Constant scale.

**Figure 2:**
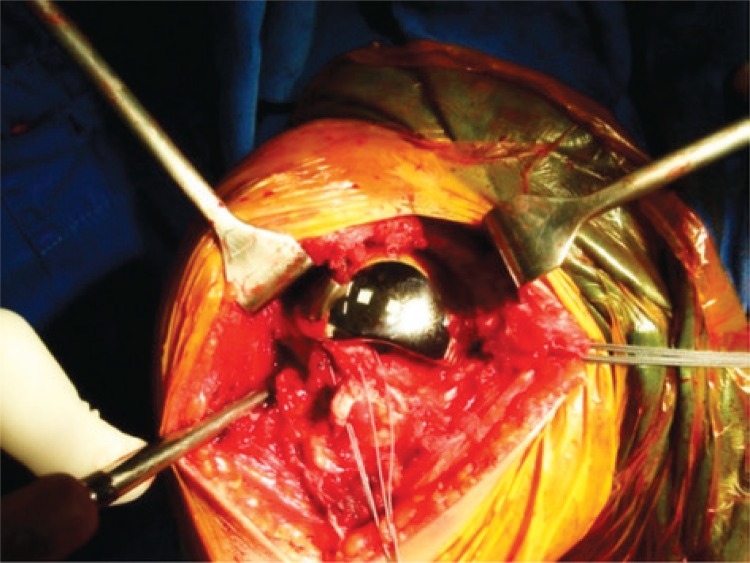
Intraoperative image of the prosthesis after implant

**Figure 3:**
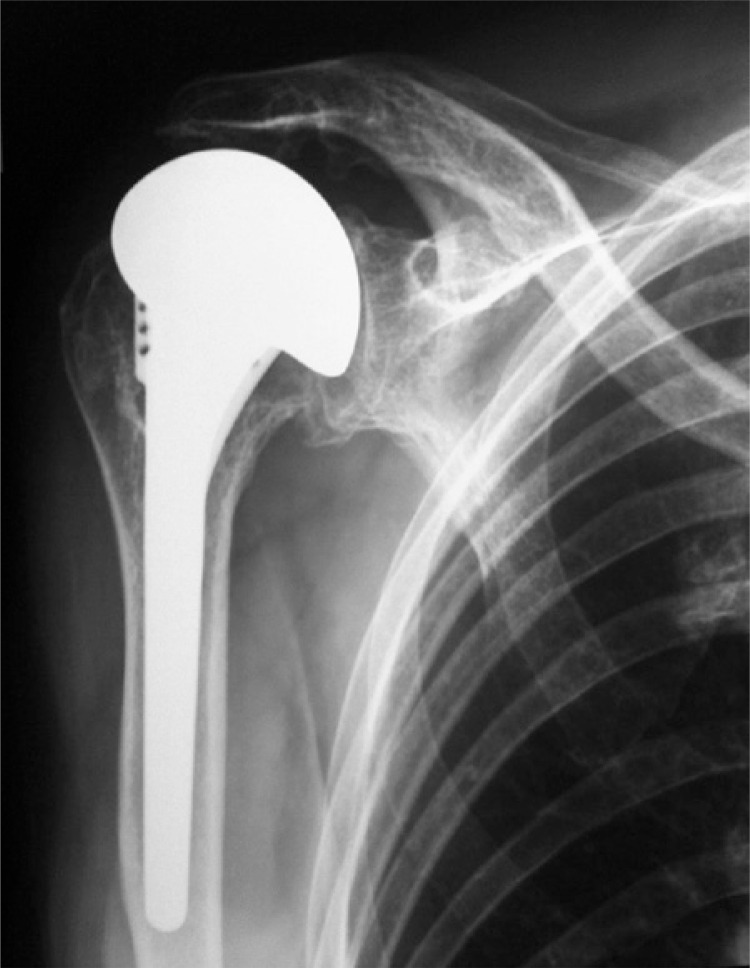
Postoperative anteroposterior radiograph

The surgical technique used can be described as follows: the patient was positioned on a beach chair, under general anesthesia, and with brachial plexus block. A deltopectoral incision was made, followed by access through the deltopectoral, disinsertion of the tendon of the subescapular muscle with repair using non-absorbable sutures, tenotomy and tenodesis of the long head of the biceps, as well as osteotomy of the humeral head with a guide for prosthesis. Moreover, the following procedures were performed: osteotomy of the greater tuberosity, rasping of the humeral canal, placement of partial press-fit prosthesis as per the recommended technique, and finally, visualization under radioscopy (Figures 2 and 3).

The variables were analyzed using the pertinent descriptive measurements: mean, standard deviation, median, minimum and maximum values for the quantitative variables; absolute (n) and relative (%) frequency for the categorical variables.

The paired *t* test was applied in the comparison between the measures of the scales in the pre- and postoperative measurements of the total sample. The variations among the pre- and postoperative scales were calculated by the absolute difference between them (before and after scales), taking into consideration sex, age group, and Seebauer classification of patients.

In the comparative analysis among the groups of interest as to the variations in scales, Student’s *t* test was used. A significance level of 0.05 (≤5%) was adopted for all statistical tests, and the Statistical Package for Social Science (SPSS) software, version 15.0 for Windows, was used for all statistical analyses.

All patients signed the Informed Consent Form. This study was submitted to evaluation and approval by the Ethics in Research with Humans Committee of the *Universidade Federal de São Paulo*, under official opinion number 503.608, CAAE: 25351113.4.0000.5505.

## RESULTS

All patients reported pain and marked functional limitation in the preoperative period. According to Seebauer classification, five patients were classified as IA, 12 as IB, eight as IIA, and nine as IIB.

No statistically significant difference was found between female and male in the means of VAS reduction (p=0.5480), as well as in the means of Constant scale increase (p=0.2451).

Considering the results obtained in age stratification, we used as cutoff the age of 75 years. In this study, 19 patients were aged over 75 years, and 15 patients were under 75 years. No statistically significant difference was found between the age groups in the means of VAS scale reductions (p=0.9199), or in the means of Constant scale increase (p=0.3447).

There was a statistically significant variation between the pre- and postoperative evaluations of VAS (p<0.0001), with a mean reduction of 6.6 points (standard deviation of 1.3 point), varying between reductions of 3 to 9 points. On the Constant Scale, there was also a statistically significant variation between the pre- and postoperative evaluations (p<0.0001), with a mean increase of 24.1 points (standard deviation of 6.4 points), varying between 13 and 39 points (Table 2).

**Table 2:** Pre- and postoperative scales in the total sample

Scales (n=34)	Evaluation		Difference	
	
	Preoperative	Postoperative	(postop minus preop)	p value
VAS				
Mean (SD)	7.7 (1.0)	1.2 (0.9)	-6.6 (1.3)	<0.0001
Median	8.0	1.0	-7	
Minimum/maximum	6/9	0/3	-9/-3	
Constants Scale				
Mean (SD)	31.5 (14.8)	55.6 (16.1)	24.1 (6.4)	<0.0001
Median	35.0	60.0	23.5	
Minimum/maximum	2/60	15/83	13/39	

Paired *t* test; VAS: Visual Analog Scale; SD: standard deviation.

**Figure 4:**
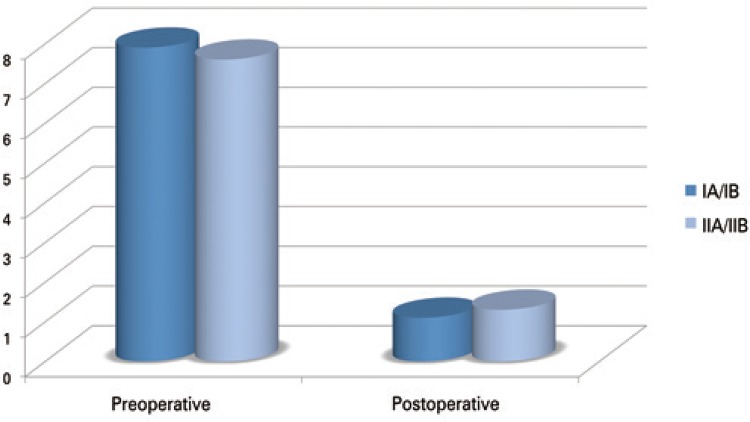
Evaluation by the Visual Analog Scaleof pain, based on the mean values found in the pre- and postoperative periods

**Figure 5:**
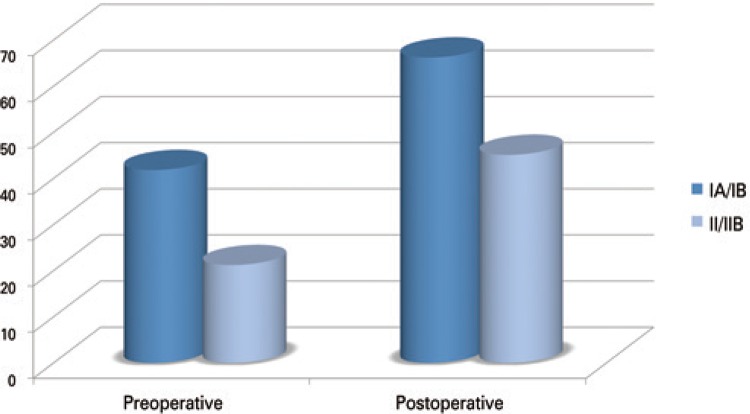
Evaluation by the Constant scale, based on the mean value found in the pre- and postoperative periods

No statistically significant difference was found between the Seebauer classification groups as to the means of VAS reduction (p=0.2348) or the means of Constant scale increase (p=0.7930) (Table 3) (Figures 4 and 5).

**Table 3:** Scales in the pre- and postoperative evaluations, as per the Seebauer classification of patients

Evaluation	VAS		Constant Scale	
	
	Seebauer Classification		Seebauer Classification	
	
	IA/IB	IIA/IIB	IA/IB	IIA/IIB
**(n=17)**	**(n=17)**	**(n=7)**	**(n=17)**
Preoperative				
Mean (SD)	7.9 (0.9)	7.6 (1.1)	41.8 (7.5)	21.2 (13.1)
Minimum – maximum	6/9	6/9	30/60	2/40
Postoperative				
Mean (SD)	1.1 (1.0)	1.3 (0.8)	66.2 (7.4)	45.1 (15.6)
Minimum – maximum	0/3	0/3	55/83	15/70
Variation (postop minus preop)				
Mean (SD)	-6.8 (1.1)	-6.3 (1.4)	24.4 (6.4)	23.8 (6.6)
Median	-7.0	-6.0	23.0	24.0
Minimum/maximum	-8/-5	-9/-3	14/39	13/35
p value	0.2348		0.7930	

Student’s *t* test; VAS: Visual Analog Scale; SD: standard deviation.

**Figure 6:**
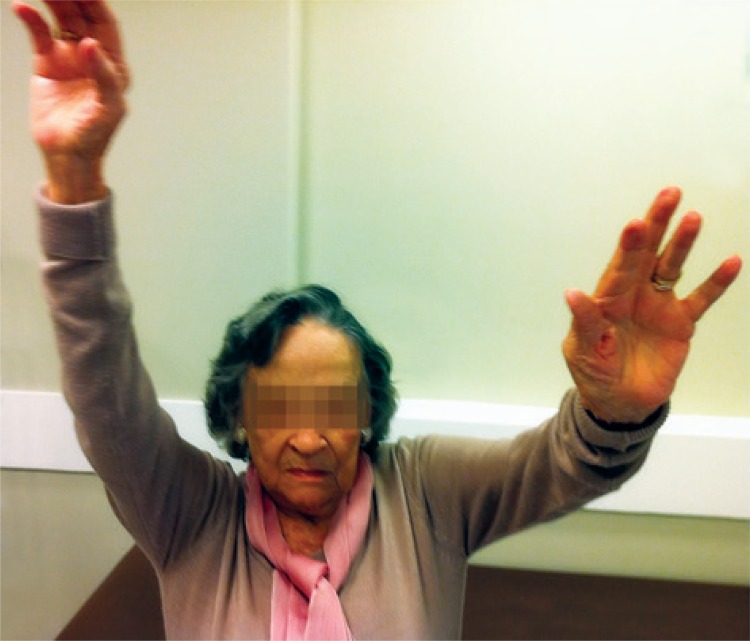
Range of motion in a patient at 2 years of postoperative follow-up

Patients preoperative with range of motion greater than 90°, Seebauer I and IIA, recovered their range of motion (Figure 6). Among patients with shoulder paralysis and Seebauer IIB, two recovered dynamic range of movement greater than 90°, two up to 90°, and four individuals remained with range of motion of less than 90°, with relief of pain. In the eight IIB cases, the patients showed pain relief. There was no statistical difference between sex, age, and the results of VAS and the Constant scale. There was no case of infection.

## DISCUSSION

The pain and functional loss resulting from cuff lesions can evolve well, from the smallest, when treated with repair, to the irreparable, treated with debridement and other surgical procedures. However, if the joint surface is affected, these procedures alone will likely not lead to a good result, and arthroplasty will be needed.

Cuff tear arthroplasty is used when the arthropathy has not affected the anterosuperior stability of the glenohumeral joint, with partial erosion of the glenoid and an intact coracoacromial arch. It is a partial prosthesis with a humeral head that extends to the greater tubercle, in order to provide contact with the coracoacromial arch, allowing a better lever arm of the deltoid muscle in arm elevation movements.^(1)^

Cuff tear arthroplasty hemiarthroplasty needs anterior stabilizers that impede anterior subluxation of the prosthesis, a role played by the coracoacromial arch and by the subscapular muscle. Additionally, it needs an intact functioning motor, such as the deltoid muscle and the axillary nerve.^(1)^ Among 16 patients submitted to hemiarthroplasty for treatment of arthropathy due to lesion of the rotator cuff, Field et al.^(9)^ found that four out of six patients with unsatisfactory results had been submitted to previous acromioplasty, with release of the coracoacromial ligament.

The poor results of total arthroplasty for treatment of rotator cuff arthropathy related to loss of the glenoid component led to hemiarthroplasty being the procedure of choice for treatment of this condition during some time. Recently, reverse shoulder prosthesis has gained great popularity due to a clinical impression of better results, despite the fact that there are still few comparative results.^(10)^

The present study observed very good results. In the total sample, there was reduction of pain, represented by a decrease, on average, of 6.6 points (varying from 3 to 9 points) on the VAS scale. Also noted was a functional improvement, with mean variation on the Constant scale of 24.1 points (varying from 13 to 39 points). From this perspective, the result of this project matches the data found in literature that shows good results with the use of hemiarthroplasty as treatment of this condition. Williams et al., reported 86% of very good results;^(11)^Field et al., 63%;^(9)^ Zuckerman et al., demonstrated 87% of satisfaction among patients;^(12)^ Sanchez-Sotelo et al. found 67% of very good results;^(13)^ and Goldberg et al., 76%.^(14)^

Nevertheless, the risk of glenoid reabsorption and movement limitation are concerns inherent to this procedure when indicated for rotator cuff arthropathy.^(5)^Reabsorption of the glenoid cavity was found in 22% and 38% of patients that presented with acromioclavicular erosion.^(13)^ Such complications were not observed in our study since they are late complications.

Brasil Filho et al.^(1)^ and Sanchez-Sotelo et al.^(13)^ demonstrated that hemiarthroplasty has an adequate level of pain relief, but only a moderate gain of functional movement in patients with rotator cuff arthropathy, corroborating the results of our study.

Arntz et al.^(15)^ compared pain in the the pre- and postoperative periods, and found that in the preoperative period, 14 of the 18 patients were classified as having incapacitating pain. In the postoperative period, three patients became pain-free, eight presented with a small intensity of pain, four had pain only after non-usual activities, and three, who had incapacitating pain, evolved with moderate pain. These results are consistent with those of our study, which observed a significant relief of pain. In the present study, also noted was an improvement of active extension, from 66° to 112°, on average; external rotation evolved from 24° to 36°, on average. Despite the fact that in radiographic evaluation all patients evolved with reduced joint space, only one progressed with erosion of the upper half of the glenoid.

Marked pain relief and improved movement were reported in the literature^(1,12,13,15)^ the latter to a smaller degree relative to the former, albeit, also significant. In patients with a very advanced stage (Seebauer IIB) for whom preferentially reverse prosthesis is indicated, a significant pain relief and less expressive range of motion were noted.

In cases classified as Seebauer IIB, CTA^®^ was chosen as treatment option due to some individualities of these patients, such as a significant involvement of the glenoid, precarious bone reserve due to osteoporosis, and associated comorbidities, since it is a procedure with less morbidity and that provides quicker benefit to this group of patients.

Our analysis demonstrated no significant difference between groups stratified by the Seebauer classification. The criticism to our study should be made due to its relatively small sample, and for having to group Seebauer IA and IB patients, and IIA and IIB patients. The patients classified as Seebauer IA/IB (n=17) obtained a 6.8-point improvement, on average, on the VAS scale, and of 24.4 points, on the Constant scale. On the other hand, the IIA/IIB (n=17) patients improved by an average of 6.3 and 23.8 points, respectively, on the VAS and the Constant scale. These values found are not statistically significant among the Seebauer groups, suggesting that the marked improvement noted in pre- and postoperative periods are similar, regardless of the severity of the joint lesion seen on the radiographs.

In 50% of patients classified as Seebauer IIB, evolution of the range of motion was less than 90°. Nevertheless, the percentage of Seebauer IIA patients that progressed with this limitation was very similar, and there was no statistically significant difference between these two groups. Despite the sample being small, it is suggested that for both cases the use of hemiarthroplasty can produce a satisfactory result, especially if the greater objective of the patient is relief of pain, since in most cases, elderly patients have a low functional requirement. Therefore, in the evaluation of the results between hemiarthroplasty and reverse prosthesis, perhaps it is more important to select well the patients to be operated on and understand their true expectations regarding the procedure.

As to the age and sex of patients, no statistical relevance was observed in the results found in those under or over 75 years of age, nor between female and male patients, suggesting that age and sex are not predictive factors as to the final results.

Reverse prosthesis has been preferred relative to hemiarthroplasty in patients with little function of the rotator cuff, since it shows better results.^(16)^ Recently however, more results in favor of hemiarthroplasty have been presented by authors that used more modern prostheses for hemiarthroplasty,^(2,13,14,17,18)^ leading some of them to elect this procedure as the treatment of choice in selected patients.^(14)^

Clinical^(19,20)^ and biomechanical^(21,22)^ studies suggested a superiority in early functional results of reverse prosthesis, but we cannot ignore the choice of hemiarthroplasty, especially when there is a considerable glenoid bone loss, since apparently, the glenoid component of reverse prosthesis becomes less safe.^(10)^ Most authors still use both procedures for treatment of these conditions, and numerous algorithms have been drawn up for treatment^(2,5,10,23,24)^ Lo et al., did not demonstrate any statistical difference as to the quality of life in patients submitted to total prosthesis and to hemiarthroplasty, despite displaying better results in total arthroplasty.^(25)^

The indication for partial arthroplasty leads to satisfactory results when we analyze all aspects involved: incapacitating pain, functional loss, intact deltoid muscle, loss of glenohumeral joint surface, loss of glenohumeral space, superior deviation of the humeral head with an intact coracoacromial arch and spherical humeral head, and irreparable deficit of the cuff, including evaluation of the patient’s expectations regarding the procedure.^(15)^

We consider the absence of a control group a limiting factor of our study. However, a control group with reverse arthroplasty in patients with comorbidities would increase operative time and the morbidity of the procedure in patients with increased risk factors. Another important limiting factor was the follow-up time, which did not take into consideration possible long-term complications inherent to the procedure.

## CONCLUSION

Shoulder hemiarthroplasty of the cuff tear is an option in patients with arthropathy of the rotator cuff, especially regarding pain complaint. Patients with an elevation greater than 90° in the preoperative phase are most benefited.

Despite the fact that more studies are necessary, with larger samples and longer periods of follow-up, there seems to be no difference in results between male and female patients, patients older than 75 years, and especially, among the different Seebauer stages. All patients of these different groups presented with significant improvement both of pain and function.
